# Expression and Functions of Fibroblast Growth Factor 10 in the Mouse Mammary Gland

**DOI:** 10.3390/ijms14024094

**Published:** 2013-02-18

**Authors:** Yingjun Cui, Qingzhang Li

**Affiliations:** Key Laboratory of Dairy Science of Ministry of Education, Northeast Agricultural University, Harbin 150030, China; E-Mail: yingjuncui2011@gmail.com

**Keywords:** mouse, mammary gland, FGF10

## Abstract

Fibroblast growth factor 10 (FGF10) is important as a mesenchymal mediator of epithelial growth and morphogenesis. In this study, the expression and localization of the FGF10 protein were detected by laser scanning confocal microscopy during mouse postnatal mammary gland development. Mammary explants were cultured to investigate the functions of FGF10. The results revealed that FGF10 localizes mainly in the mesenchyme near the ductal epithelial cells and the alveolar epithelial cells of the mammary gland. Peak FGF10 expression levels were observed at lactation day 10. FGF10 induced FGFR2-IIIb expression in the mammary epithelium, except in virgin or pregnant mice. FGF10 promoted the proliferation of mammary gland epithelial cells and reduced apoptosis. FGF10 is important during the mouse mammary gland growth, development, and reconstruction, and its effects are mediated by FGFR2-IIIb.

## 1. Introduction

The fibroblast growth factor (FGF) family comprises at least 22 members, many of which have been implicated in multiple aspects of vertebrate development. In particular, fibroblast growth factor 10 (FGF10) has been associated with branching morphogenesis [1]. The cDNA encoding FGF10 was isolated and named in 1996 [2]. It is highly expressed in the lung and at lower levels in the heart, skin, pancreas and brain [3]. Two types of FGF receptors (FGFRs) bind to FGF10, FGFR2-IIIb and FGFR1-IIIb. FGF10 binds to FGFR2-IIIb with high affinity [4].

FGF10 can potentially signal to the mammary epithelium in a paracrine manner. Development of the mouse mammary gland occurs in three separate stages: embryonic, postnatal and post-pubertal [5]. During embryogenesis, the intra-somitic FGF10 gradient, together with ventral elongation of the somites, determines the correct dorsoventral position of the mammary epithelium along the flank [2,6]. In addition, signaling by the FGF10 receptor FGFR2b involved in mammogenesis is critical not only during embryogenesis but also during postnatal development to allow the formation and maintenance of the terminal end buds and to control the survival and proliferation of the luminal epithelial progenitor cells [7,8]. Moreover, *FGFR2* mRNA expression levels are eight times higher in the tumor-derived fibroblasts of human female breast cancer patients than in the corresponding fibroblasts from normal breast tissue [9].

No information is available concerning the dynamics of FGF10 protein expression or function in the mammary gland. The aim of this descriptive study is to examine the dynamic pattern of protein expression and localization of FGF10 during postnatal mammary gland development. Furthermore, the function of FGF10 will be investigated during defined mouse mammary gland developmental stages. The data are intended to direct more experimentally oriented studies to find the new methods for exogenous control of lactation levels.

## 2. Results

### 2.1. Expression and Localization of FGF10 in the Mouse Mammary Gland

**Figure 1:**
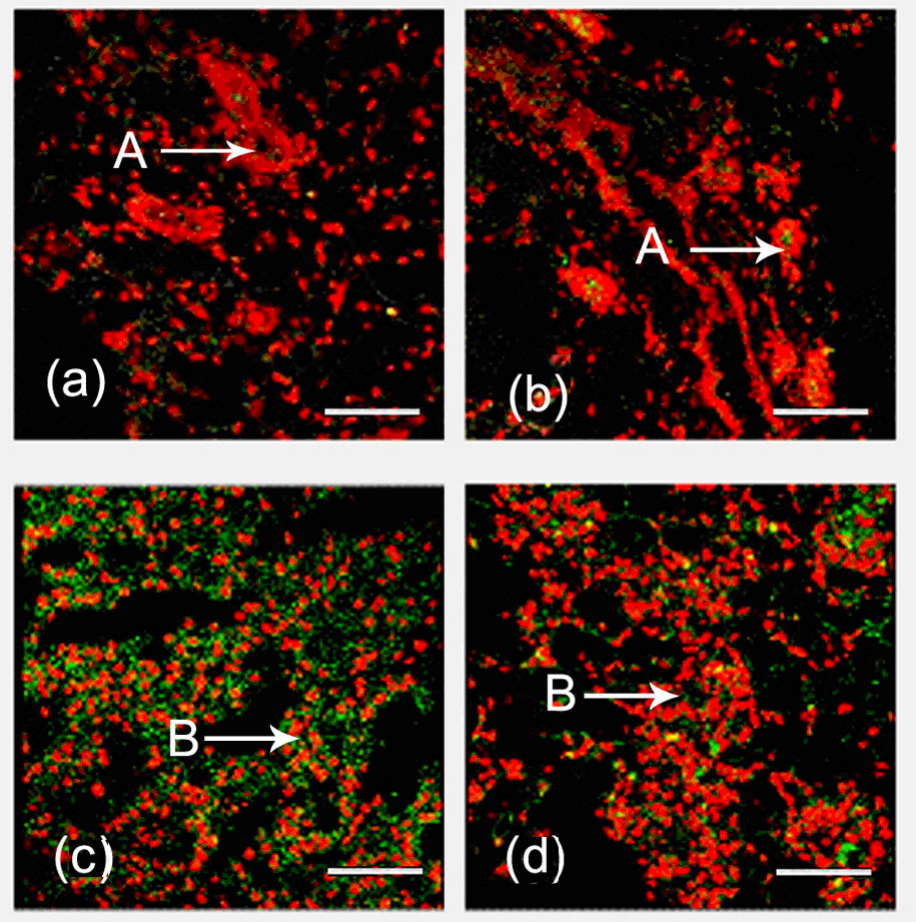
Localization of FGF10 in the mouse mammary gland. Confocal microscopic images are shown. FGF10 localization is shown in virgin day 50 (**a**); pregnancy day 4 (**b**); lactation day 10 (**c**) and involution day 4 (**d**). The localization of FGF10 (green) was detected using an indirect immunofluorescence technique. The cell nucleus (red) was stained with propidium iodide (PI). “A” in each picture indicates ductal epithelial cells; “B” in each picture indicates alveolar epithelial cells. (400×). Bars, 50 μm.

**Figure 2:**
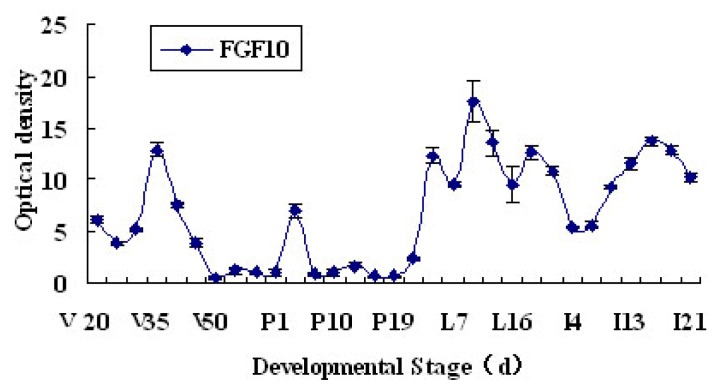
FGF10 expression levels in the mouse mammary gland. “V”, “P”, “L”, and “I” indicate virgin, pregnancy, lactation and involution, respectively.

Immunofluorescence was used to reveal the localization of FGF10. During the development of the mouse mammary gland, FGF10 localized mainly in the mesenchyme near ductal epithelial cells and/or the mesenchyme near alveolar epithelial cells (Figure 1). The fluorescence optical density, analyzed by using Image Pro-Plus 5.0, indicates the level of FGF10 expression. The expression of FGF10 fluctuated during the study period. Higher expression levels were observed in virgin day 20 (*p* < 0.05), virgin day 35 (*p* < 0.05), pregnancy day 4 (*p* < 0.05), and lactation day 10 (*p* < 0.05). FGF10 expression levels declined dramatically in involution day 4 (*p* < 0.05) and involution day 7 (*p* < 0.05), then increased and remained high. Peak expression of FGF10 was detected in lactation day 10 (Figure 2).

### 2.2. Effects of FGF10 in the Mouse Mammary Gland

**Figure 3:**
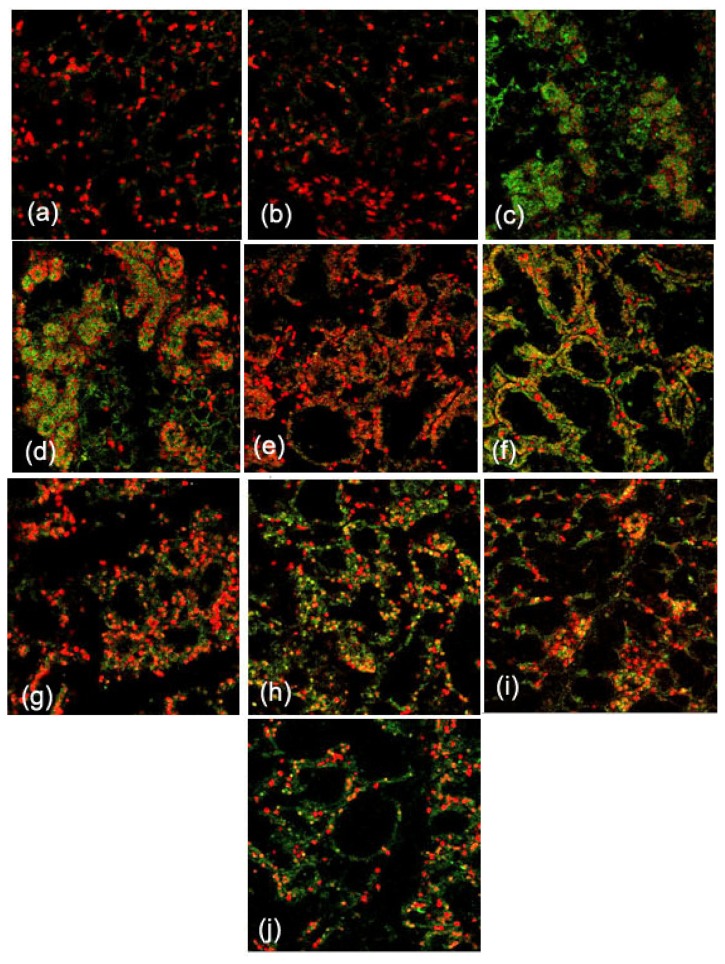
The localization of FGFR2-IIIb in mouse mammary explants after culture for 24 h. FGFR2-IIIb expression is shown in mammary explants from mice during virgin (**a**), pregnancy (**c**), lactation (**e**), forced involution (**g**), and involution (**i**) and cultured without FGF10 treatment. FGFR2-IIIb expression is shown in mammary explants prepared from mice during virgin (**b**), pregnancy (**d**), lactation (**f**), forced involution (**h**), and involution (**j**) and cultured with FGF10 treatment. The localization of FGFR2-IIIb (green) was detected using an indirect immunofluorescence technique. The cell nucleus (red) was stained with propidium iodide (PI) (400×).

**Figure 4:**
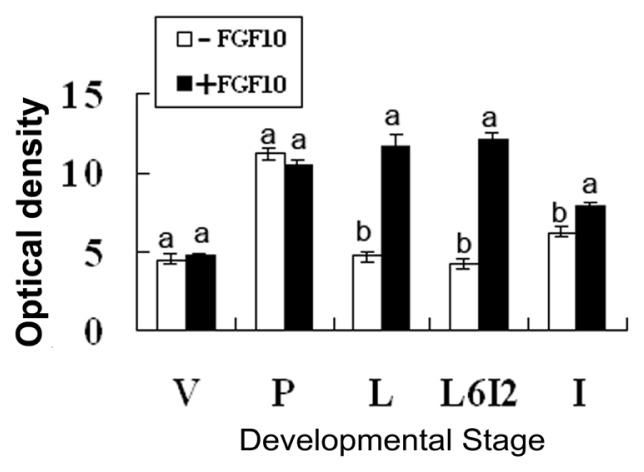
FGFR2-IIIb expression levels in mouse mammary explants after culture for 24 h. “V”, “P”, “L”, “L6I2”, and “I” indicate explants from mice during virgin, pregnancy, lactation, forced involution and involution, respectively. Each bar represents the Mean ± SD. Bars with different letters are significantly different (*p* < 0.05).

Frozen sections were prepared from mammary explants treated with or without 50 ng/mL FGF10 for 24 h. FGFR2-IIIb expression levels in the mouse mammary explants were measured by immunofluorescence (Figure 3). Fluorescence optical density of all the photos was analyzed by using Image Pro-Plus 5.0. FGF10 treatment induced FGFR2-IIIb expression in the mammary epithelium during lactation, forced involution, and involution (*p* < 0.05; Figure 4).

**Figure 5:**
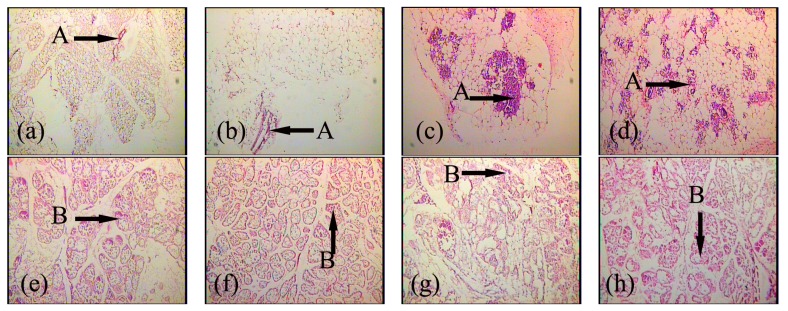

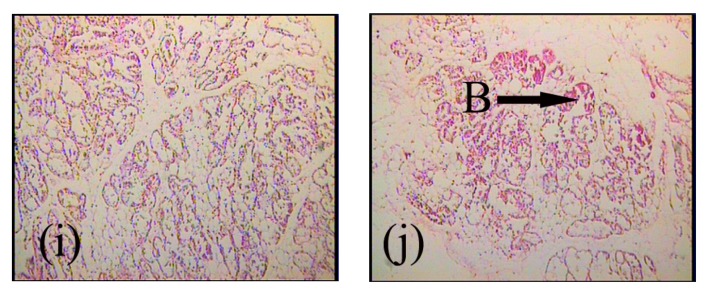
Paraffin sections of the mouse mammary gland after explant culture for 120 h. Paraffin section images of mammary explants from mice during virgin (**a**); pregnancy (**c**); lactation (**e**); forced involution (**g**), and involution (**i**) and cultured without FGF10 treatment are shown. Paraffin section images of mammary explants from mice during virgin (**b**); pregnancy (**d**); lactation (**f**); forced involution (**h**) and involution (**j**) and cultured with FGF10 treatment are shown. “A” in each picture indicates the duct; “B” in each picture indicates the acinus (100×).

**Figure 6:**
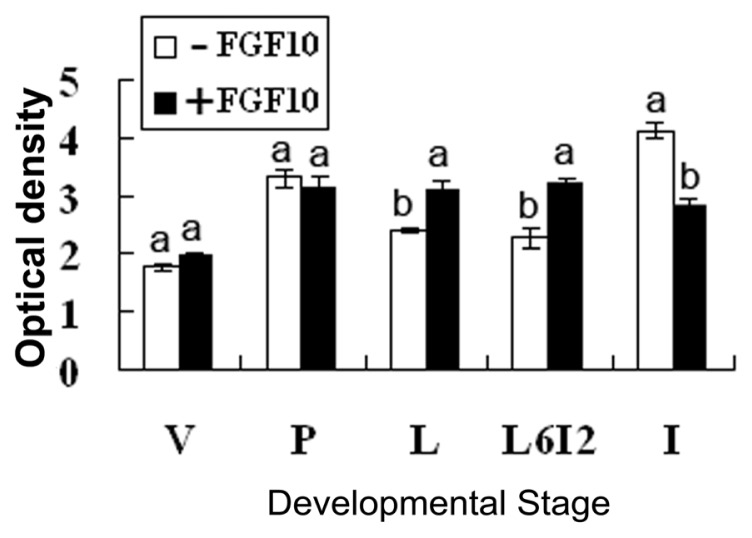
Development of the mouse mammary epithelium after explant culture for 120 h. “V”, “P”, “L”, “L6I2”, and “I” indicate explants from mice during virgin, pregnancy, lactation, forced involution, and involution, respectively. Each bar represents the Mean ± SD. Bars with different letters are significantly different (*p* < 0.05).

Paraffin sections were prepared from mammary explants treated with or without 50 ng/mL FGF10 for 120 h. Morphology was evaluated by analyzing the color segmentation of the paraffin section images. The FGF10-treated lumen of the mammary duct is bigger than the control group in virgin mice (Figure 5b), and FGF10 promoted mammary ductal branching during pregnancy (Figure 5d). However, the quantity of mammary gland epithelial cells did not increase during either stage (*p* > 0.05). FGF10 increased the quantity of mammary gland epithelial cells during lactation (*p* < 0.05; Figure 5f), maintained the alveolar morphology, and increased the quantity of mammary gland epithelial cells during forced involution (*p* < 0.05; Figure 5h). FGF10 maintained the intact morphology of the acinus but did not increase the quantity of mammary gland epithelial cells during involution (Figures 5j and 6).

**Figure 7:**
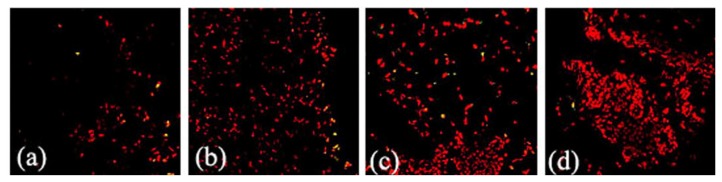

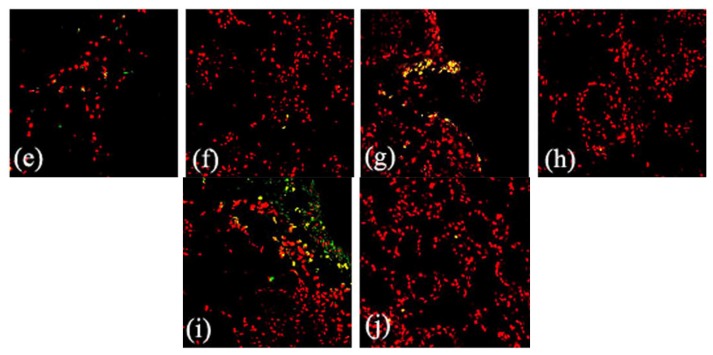
Apoptosis in the mouse mammary gland after explant culture for 24 h. Images illustrating apoptosis in mammary explants prepared from mice during virgin (**a**); pregnancy (**c**); lactation (**e**); forced involution (**g**), and involution (**i**) and cultured without FGF10 treatment are shown. Images illustrating apoptosis in mammary explants prepared from mice during virgin (**b**); pregnancy (**d**); lactation (**f**); forced involution (**h**), and involution (**j**) and cultured with FGF10 treatment are shown. The cell nucleus (red) was stained with propidium iodide (PI) (400×).

**Figure 8:**
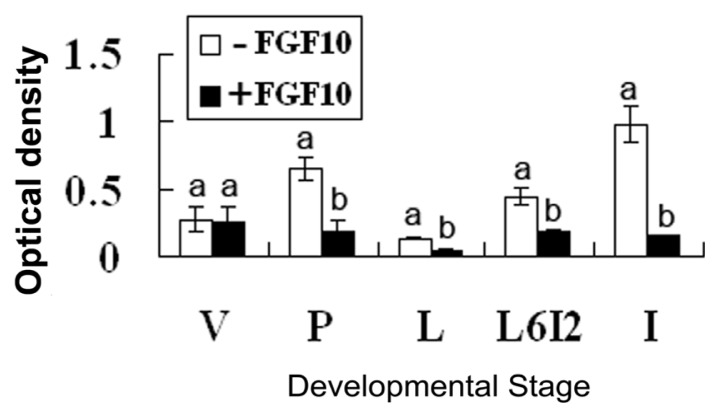
Levels of apoptosis in the explants of mouse mammary gland. “V”, “P”, “L”, “L6I2”, and “I” indicate explants prepared from mice during virgin, pregnancy, lactation, forced involution, and involution, respectively. Each bar represents the Mean ± SD. Bars with different letters are significantly different (*p* < 0.05).

Apoptosis was assayed using the Dead End™ Fluorometric TUNEL System. Frozen sections were prepared from mammary explants treated with or without 50 ng/mL FGF10 for 24 h. Fluorescence optical density of all the photos was analyzed by using Image Pro-Plus 5.0. FGF10 treatment reduced levels of apoptosis in all mammary gland explants except those of virgin mice (*p* < 0.05; Figures 7 and 8).

## 3. Discussion

### 3.1. Expression and Localization of FGF10 in the Mouse Mammary Gland

FGF10 is highly expressed in branched epithelial organs such as the mouse lung [10] and prostate [11]. FGF10 is expressed in the mesenchyme near the distal tips of epithelial lung buds [12]. Similarly, using an indirect immunofluorescence technique, we showed that FGF10 is localized in the mesenchyme near mammary ductal epithelial cells and in the mesenchyme near the mammary alveolar epithelial cells.

We detected high levels of FGF10 expression in the mammary gland of 20-day-old virgin mice. The high FGF10 expression at this stage might be related to the early development of the mammary gland. In virgin mice older than 35 days, the expression of FGF10 began to decline except during a short-term period of high expression at pregnancy day 4. Low levels of expression are maintained until the end of pregnancy. The short-term high expression observed at pregnancy day 4 might be related to the formation and branching of the mammary duct. The expression of FGF10 began to increase during lactation and peaked at lactation day 10; subsequently, FGF10 expression began to decline during early involution, but then increased and remained high after involution day 10. The increased FGF10 expression during lactation might be related to mammary structure maintenance, and the expression of FGF10 during involution might be related to the development of adipose tissue and mammary gland reconstruction.

### 3.2. FGF10 and Mouse Mammary Gland Morphology

FGF10 promotes the proliferation, differentiation, and migration of epithelial cells. FGF10 signaling regulates branching during the early organ development [13]. Deletion of the FGF10 gene completely prevents limb bud formation and causes serious defects in lung branching [14,15]. To address the relationship between the expression and functions of FGF10 in the mouse mammary gland, we compared the expression of FGFR2-IIIb and the morphology of the mammary gland with or without FGF10 treatment. FGF10 failed to induce mammary gland epithelial cells to produce FGFR2-IIIb in virgin mice because the mammary gland epithelial cells were few in number. FGF10 treatment did not change the quantity of mammary epithelial cells in virgin mice, either. However, FGF10 treatment did enlarge the lumen of the mammary duct and cause visible morphological changes. Although expression of FGFR2-IIIb and the quantity of mammary gland epithelial cells in the FGF10-treated group did not change significantly compared to the control group during pregnancy, paraffin sections showed that FGF10 increases ductal branching within the mammary gland during this period. This observation suggests that the mammary gland resists FGF10 induction of FGFR2-IIIb during pregnancy. This resistance might be related to duct branching. FGF10 caused significant induction of FGFR2-IIIb in the mammary epithelium during lactation, forced involution, and involution. At the same time, FGF10 treatments during these three stages revealed its functions. FGF10 increased the number of mammary gland epithelial cells during lactation, increased the number of mammary gland epithelial cells during forced involution, and maintained the complete morphology of the acinus of the mammary gland during forced involution and involution but did not increase the number of mammary gland epithelial cells during involution.

### 3.3. FGF10 and Apoptosis

Apoptosis is controlled by the ratio of various Bcl-2 family members; certain members promote cell survival (Bcl-2, Bcl-xl, and Bcl-w) while others promote cell death (Bax, Bad, Bak, and Bim) [16]. The activation of FGFRs is involved in cell survival through downstream signaling cascades such as the mitogen-activated extracellular regulated kinase kinase (MEK)/extracellular regulated kinase (ERK) and the phosphatidylinositol 3-kinase/protein kinase B pathways. These signals influence survival through several mechanisms including the regulation of Bcl-2 and its family members [17]. Recent research revealed that FGF10 decreases levels of apoptosis in part by mechanisms involving MEK/ERK-dependent signaling that affect the mitochondria-regulated death pathway [18]. In this report, we also found that FGF10 reduced levels of apoptosis in the mammary gland, but the mechanism by which this occurs needs further study.

## 4. Materials and Methods

### 4.1. Animals, Tissue Sampling, and Preparation

Female Kunming mice (Harbin Medical University) were maintained and bred under controlled temperature and lighting. All animals received humane care as outlined in the Guide for the Care and Use of Experimental Animals. In separate experiments, inguinal mammary glands were cut into small pieces (1–2 g) and immediately frozen in liquid nitrogen for frozen section. Frozen sections (8–10 μm) were mounted onto slides coated with 3-triethoxysilylpropylamine and stored at 4 °C.

Animals were classified into the following groups and subgroups, where the subgroup nomenclature is comprised of a letter that represents the group and the day at which the mice of the subgroups were sacrificed for study: (i) Virgin mice, including V20 d, V25 d, V30 d, V35 d, V40 d, V45 d, V50 d, V55 d, and V60 d; *n* = 3 for all subgroups, (ii) pregnant mice, including P1 d, P4 d, P7 d, P10 d, P13 d, P16 d, P19 d; *n* = 3 for all subgroups, (iii) lactating mice, including L1 d, L4 d, L7 d, L10 d, L13 d, L16 d, L19 d; *n* = 3 for all subgroups, (iv) mice during involution, including I1 d, I4 d, I7 d, I10 d, I13 d, I16 d, I19 d, I21 d; *n* = 3 for all subgroups.

To prepare mammary explant cultures, the inguinal mammary glands were excised from the subgroups V30 d (*n* = 3), P12 d (*n* = 3), L12 d (*n* = 3), L6I2 d (forced involution, remove litters from lactating mice at L6 d, then obtain the tissues after another 2 days) (*n* = 3).

### 4.2. Materials

FGF10 antibody (H-121) (sc-7917), Bek antibody (C-17) (sc-122), and fluorescein isothiocyanate (FITC)-conjugated goat anti-rabbit IgG (sc-2012) were purchased from Santa Cruz Biotechnology. Recombinant human FGF10 was purchased from R&D Systems. Waymouth’s MB752/1 was purchased from Gibco. Insulin, prolactin, hydrocortisone, DABCO (1,4-diazobicyclo(2,2,2)octane), and PI were purchased from Sigma. The Dead End™ Fluorometric TUNEL System was purchased from Promega.

### 4.3. Mammary Explant Culture

The inguinal mammary glands were cut into 2–3 mm^3^ pieces and one piece was inoculated into each well of a 6-well plate in Waymouth’s MB752/1 medium with 5 μg/mL hydrocortisone, 5 μg/mL insulin, 5 μg/mL prolactin, and with or without 50 ng/mL FGF10. Explants were grown at 37 °C in a 5% CO_2_–95% air atmosphere. The medium was changed every other day.

The explants to be used for the analysis of FGFR2-IIIb expression and apoptosis were frozen after culture for 24 h. The explants used to prepare paraffin sections for morphological analysis were cultured for 120 h.

### 4.4. Immunofluorescence

The presence of FGF10 and its receptor was demonstrated by an indirect immunofluorescence technique. Non-specific protein binding was prevented by incubation with 10% normal goat serum in phosphate-buffered saline (PBS) for 2 h at room temperature. Sections were then incubated overnight at 4 °C with a rabbit polyclonal antibody to FGF10 or FGFR2-IIIb (diluted 1:100), for 30 min with FITC-conjugated goat anti-rabbit IgG (diluted 1:50 in 1% bovine serum albumin in PBS), and for 5 min at room temperature with 1 μg/mL PI in PBS. DABCO was used to mount the sections. Between each step, the sections were washed three times in PBS. All incubations were carried out in humidified chambers to prevent evaporation. Negative controls were incubated with rabbit IgG instead of the primary antibody.

### 4.5. Apoptosis

Apoptosis was assayed with the Dead End™ Fluorometric TUNEL System. After culture for 24 h, frozen sections were prepared from the explants. The frozen sections were fixed by immersing slides into freshly prepared 4% methanol-free formaldehyde in PBS, pH 7.4, in a Coplin jar and incubating for 25 min at 4 °C. The slides were washed twice by immersion into fresh PBS for 5 min at room temperature. The frozen sections were permeabilized by immersing the slides into 0.2% Triton X-100 in PBS for 5 min. The slides were rinsed twice by immersion into fresh PBS for 5 min at room temperature. Excess liquid was removed by tapping the slides. The frozen sections were covered with 100 μL Equilibration Buffer and equilibrated at room temperature for 5–10 min. Nucleotide Mix was thawed on ice and a sufficient amount of rTdT incubation buffer for all the experimental reactions and positive controls was prepared. For negative controls, a control incubation buffer without rTdT Enzyme was prepared by combining 45 μL Equilibration Buffer, 5 μL Nucleotide Mix, and 1 μL autoclaved, deionized water. For the experimental reactions and positive controls, an incubation buffer was prepared by combining 45 μL Equilibration Buffer, 5 μL Nucleotide Mix, and 1 μL rTdT Enzyme. The positive controls were pretreated by DNase I before this step. The positive control slides were processed in separate Coplin jars during subsequent steps. Tissue paper was used to blot around the equilibrated areas and 50 μL incubation buffer was added to a 5 cm^2^ area of the frozen sections. The frozen sections were covered with plastic coverslips to ensure even distribution of the reagent. The slides were incubated at 37 °C for 60 min inside a humidified chamber. The plastic coverslips were removed and the reaction was terminated by immersing the slides into 2× saline sodium citrate buffer for 15 min at room temperature. The samples were washed three times by immersing the slides into fresh PBS for 5 min at room temperature. The samples were stained by immersing the slides into PI solution freshly diluted to 1 μg/mL in PBS for 5 min at room temperature in the dark. The samples were washed three times by immersing the slides into deionized water for 5 min at room temperature. Excess water was drained from the slides and the area surrounding the tissues was wiped with tissue paper. The sections were mounted by DABCO.

### 4.6. Image Analysis

Each experiment was repeated three times. Each section was observed by choosing five different visual fields. The images were digitized using a charge-coupled device color video camera mounted on a laser scanning confocal microscope. Images were analyzed by using Image Pro-Plus 5.0. We measured fluorescence, defining optical density as the fluorescence intensity value divided by the area over which fluorescence was measured.

Morphology was evaluated by color segmentation analysis of the paraffin section images.

### 4.7. Statistical Analysis

All data were expressed as the mean ± standard deviation (*n* = 3) and were tested for statistical significance (*p* < 0.05) by using SPSS 16.0 (SPSS Inc.: Chicago, IL, USA). A statistical comparison of the means among the groups was performed using one-way analysis of variance. Differences between the means of individual groups were analyzed by Tukey post hoc tests. Statistical significance was declared at *p* < 0.05.

## 5. Conclusions

FGF10 plays important roles in the mouse mammary gland. These effects are mediated by FGFR2-IIIb, which is induced by FGF10. These data showed that the hormonal regulation of FGF10/FGFR2-IIIb axis potentially affects the growth, development, and reconstruction of the mouse mammary gland.
